# Determinants of adolescent substance use in Africa: a systematic review and meta-analysis protocol

**DOI:** 10.1186/s13643-021-01680-y

**Published:** 2021-04-27

**Authors:** Sandra Jumbe, Tony Mwenda Kamninga, Isaac Mwalwimba, Ukwuori-Gisela Kalu

**Affiliations:** 1 The Institute of Population Health Sciences (IPHS), Barts and The London School of Medicine and Dentistry, Queen Mary University of London, Yvonne Carter Building, 58 Turner Street, London, E1 2AB UK; 2 African Institute for Development Policy (AFIDEP), P.O Box 31024, Lilongwe, Malawi; 3 Millennium University, P.O Box 2797, Blantyre, Malawi; 4 Healios Ltd., Mountbatten Business Centre, Southampton, SO15 UK

**Keywords:** Substance use, Adolescent, Determinants, Africa, Systematic review

## Abstract

**Background:**

Adolescent substance use continues to be a growing major public health concern in Africa. Recent studies infer an overall estimated prevalence of 42% among adolescents in sub-Saharan Africa. Unfortunately, this phenomenon is not adequately documented across many settings in the continent despite known negative health and social consequences on affected individuals and their communities. Little is known about the social context of substance use in Africa among this population. Our aim is to conduct a systematic review, exploring the determinants and associated factors that influence adolescent substance use in Africa.

**Methods:**

We will search the following databases (from January 2000 onwards): PubMed, the Cochrane Library, African Journals Online (AJOL), Google Scholar, ScienceDirect and the World Health Organization (WHO) African Index Medicus. We will include population-based observational studies reporting on determinants and/or risk factors of substance use among adolescents (age 10–19 years) across Africa. Two reviewers will independently screen all citations, full-text articles and abstract data. Potential conflicts will be resolved through discussion. Study methodological quality (or bias) will be appraised using appropriate tools. If feasible, we will conduct a random-effects meta-analysis of data. We plan to conduct a meta-synthesis of qualitative studies where appropriate

**Discussion:**

This review will describe the range of determinants and associated factors found to significantly influence adolescent substance use in Africa over the last two decades. Documenting this evidence is important as it can potentially inform comprehensive interventions and treatment programmes that are targeted at adolescents and their parents in these settings.

**Systematic review registration:**

PROSPERO CRD42020190158

**Supplementary Information:**

The online version contains supplementary material available at 10.1186/s13643-021-01680-y.

## Background

Adolescents, defined by the World Health Organization (WHO) as people aged 10 to 19 years [1], represent a fifth of the global population; however, their health is often neglected because they are perceived to be healthy [2, 3]. Rates of mental, neurological and substance use disorders are highest during adolescence and early adulthood [4]. Substance use which includes alcohol, tobacco and illicit drug use is common during adolescence [5]. Young people who misuse alcohol and drugs experience more medical symptoms such as appetite changes, weight loss, headaches, sleep disturbance and depression than their counterparts [3, 6] with negative effects on learning and development [7]. Africa’s population has grown by over 50% since 2000, and in this time, the number of years lost to disability because of mental and substance use disorders increased by 52% [8]. Globally, substance use is a key cause of disability-adjusted life years (DALYs) lost in young people; DALY rates in Africa are 2.5 times higher than in high-income countries [2, 4].

Recent studies in Africa indicate a high prevalence of substance use among young people when compared to the general population, with associated physical and psychosocial problems such as fighting, vandalism, theft, engaging in unprotected sex, personal injury, medical problems and impaired relationships with family and friends [9–11]. A recently published systematic review found that the overall prevalence of ‘any substance use’ among adolescents in sub-Saharan African is 41.6%, with alcohol and tobacco being the highest prevailing substances (i.e. 40.8% and 45.6%, respectively) across the continent compared to any other substance use [12]. More interestingly, a few region-specific patterns of substance use were identified, highlighting the need for region (or country)-specific and culturally appropriate interventions and policies, for example, khat use only in East Africa [13] and using tranquilisers in South Africa [12].

Health and illness are determined by complex interactions between social and economic factors, the physical environment and individual behaviour [14]. Determinants are a range of factors that influence substance use among individuals or populations. To our knowledge, there is no systematic review that provides an accurate understanding of the determinants of adolescent substance use in Africa. Broadly speaking family, social networks and peer pressure are key influencers of substance abuse among adolescents [10, 14]. For example, several African studies indicate having family or friends who use substances is a key risk factor [10, 15, 16]. Childhood trauma and adverse experience like physical, emotional and sexual abuse is another significant risk factor for substance use [17]. Other demographic and socioeconomic risk factors have included being male [18], of younger age, lower education grades, adolescents from divorced parents, and unemployed or fully employed mothers in SA [19] and private school attendance [16]. Some differences have been noted between urban and rural adolescent populations. For urban areas in Nigeria, having friends who use substances and a mother with tertiary education are risk factors whilst parental disapproval of substance use is a protective factor [16]. Adolescent disapproval of adult substance use is a protective factor in rural areas.

Substance use among adolescents is a growing major public health concern in Africa [12]. Unfortunately, this phenomenon has not been adequately documented across many settings in the continent. Knowledge on the true extent and determinants of adolescent substance use in Africa is limited, perhaps due to socioenvironmental factors, competing health priorities and limited treatment options [3]. Moreover, little is known about the relationship between determinants of adolescent substance use in Africa. This is despite the increasing prevalence and increased risks of severe health, economic and social problems due to substance use. Even with the increased need for substance use programmes and policies in Africa highlighted in the Sustainable Development Goals (SDG 3.5), there is no consolidated evidence about the social contexts of substance use among adolescents in Africa [9]. Harmful adolescent substance use is not due to any single cause, but a rather complex interaction of risk and protective factors mainly shared with other problematic behaviours like school non-attendance or violence. A conceptual framework for determinants of substance use illustrates this multilevel complexity, by outlining how substance misuse is influenced by an individual’s development, experiences and health, as well as how they interact with their family, their community environment and broader societal-level factors like culture, policy and socioeconomic status [14]. Effectively tackling substance use among young people in Africa where negative outcomes and a complexity of unconventional determining factors for substance use are more common would bring great benefit to human health.

Existing reviews do not provide a detailed holistic and regional understanding of the determinants of substance use among adolescents in sub-Saharan Africa. As previously stated, substance use among adolescents can lead to an increased risk of transmission of sexually transmitted infections, vehicular fatalities, juvenile delinquency and other problems associated with physical and mental health [4]. Adolescents’ increased susceptibility to involvement in substance use can lead to reduced decision-making ability and increased long-term effects of drugs and alcohol [20].

The objective of this study will be to systematically evaluate qualitative and quantitative epidemiological studies describing the determinants of substance use among adolescents aged 10 to 19 years old in Africa.

## Methods/design

### Protocol registration and reporting

The present protocol has been registered within the PROSPERO database (registration number CRD42020190158) and is being reported in accordance with the reporting guidance provided in the Preferred Reporting Items for Systematic Reviews and Meta-Analyses Protocols (PRISMA-P) statement [21] (see checklist in Additional file 1).

### Information sources and search strategy

The primary source of literature will be a structured search of several electronic databases (from January 2000 to 31 December 2019—this period was selected to cover the millennium years in the hope to capture the indicated rise in substance use among this target population): PubMed/MEDLINE, the Cochrane Library, African Journals Online (AJOL), Science Direct and the World Health Organization (WHO) African Index Medicus (AIM) regional database. The secondary source of potentially relevant material will be a search of the grey or difficult to locate literature, including Google Scholar. We will perform hand-searching of the reference lists of included studies, relevant reviews, national clinical practice guidelines or other relevant documents. Content experts and authors in the field will be contacted. The literature searches will be designed and conducted by the review team which includes two experienced health information specialists. The search will include a broad range of terms and keywords related to determinants, factors, adolescent, substance use and Africa. A draft search strategy for PubMed/MEDLINE is provided in Additional file 2

### Eligibility criteria

The main outcome of this review will be to characterise the key determinants that have been found to significantly influence any substance use among adolescents in Africa. The role of other study-level variables like gender and (mental) health status as potential risk factors will also be explored with regional comparisons where possible.

#### Inclusion criteria

Studies will be included in this review based on the following criteria:

Participants: We will include studies involving adolescents aged 10–19 years old, according to the WHO definition [1, 22].

Condition/outcome of interest: The primary outcomes of interest will be determinants or factors influencing substance use. A determinant will be defined as a factor reported to decisively affect or influence adolescent substance use [14]. Potential examples include genetics, family or peer pressure. The term ‘substance use’ refers to the use of drugs or alcohol, including substances such as cigarettes, illegal drugs, prescription drugs, inhalants and solvents [12].

Study design and context: Eligible studies will be quantitative observational studies (e.g. cohort, cross-sectional or health surveys) and qualitative studies conducted in Africa, reporting information on outcome data.

#### Exclusion criteria

We will exclude non-human studies, reviews and methodological studies. No limitations will be imposed on publication status (unpublished studies will be eligible for inclusion) and language of publication.

### Screening and selection procedure

Two reviewers will independently conduct article selection electronically using the eligibility criteria. Relevant articles will initially be determined by title and abstracts, followed by full paper assessment as per the inclusion criteria, with reasons for exclusion noted at each stage. Firstly, two reviewers (UK and IM) will independently go through all resultant study titles from the database searches, remove studies that do not meet the inclusion criteria then merge and deduplicate the lists of eligible studies. The two reviewers will then meet to discuss any discrepancies in selected articles and any remaining disagreements will be adjudicated by a third reviewer (SJ). Abstract screening will be completed by SJ and TM using the same process used for title screening. UK will be the third reviewer to resolve the remaining disagreements in selected articles. A Microsoft Excel spreadsheet will be used to record the title and abstract selection process. The full paper screening process will be logged onto an electronic database. A PRISMA flow chart showing the details of studies included and excluded at each stage of the study selection process will be provided.

### Quality assessment

The methodological quality of each study will be evaluated independently by two reviewers (SJ and TM). The tool developed by the Effective Public Health Practice Project will be used to assess the quality for quantitative studies [23]. This tool has been chosen because of its ability to extensively assess methodological quality and its usability across different quantitative research designs. The tool will consider aspects like the presence of selection bias and confounders, study design, blinding, data collection methods, withdrawals and drop-outs, and the appropriateness of the study’s analysis to the research question [23, 24]. The quality of qualitative studies will be assessed using the Critical Appraisal Skills Programme (CASP) developed by the Public Health Resource Unit, National Health Service, England [25]. The CASP includes 10 questions to assess the rigorousness, credibility and relevance of the qualitative study by answering yes/no for each question. The first two questions are general screening questions that consider the clarity of the study goal, and whether the study methodology is appropriate. When both questions are positively answered, reviewers proceed to the remaining questions to consider methodological quality [24].

The two reviewers will independently assess the articles prior to inclusion in the final review using these checklists. Studies will be graded using these tools to assess the quality of the study design and reporting including information about how substance use determinants are measured and operationalised. Discrepancies in the reviewers’ evaluations will be discussed until consensus is reached. Any disagreement that arises between the reviewers’ ratings will be solved by involving a third reviewer.

### Data extraction

All reviewers will independently go through full papers of included studies to remove studies that do not meet the inclusion criteria, using a data extraction form held on a database. This will occur simultaneously with the quality assessment process. This form will include the following information: author(s) and publication date, study setting, research focus, study design, sample size, participants’ age mean/range, substance type (e.g. alcohol, tobacco), determinants (reported factors associated with substance use) and key findings. To increase concordance during this phase, all reviewers will pilot using the data extraction form by extracting data from the same 5 papers independently then discussing any disagreements/discrepancies as a team. Over time, SJ and/or UK will randomly select and cross-check the outcomes of 10 full papers previously extracted by the other reviewers to gauge consistency.

### Data synthesis

The characteristics and quality assessment of the included studies will be presented in tables. Research findings will be pooled using the data extract from the review database. Both quantitative and qualitative synthesis analyses will be used in this study.

We will conduct a narrative synthesis of the results from the included studies, focusing on the association between each determinant and substance use among adolescents in Africa. Where possible, we will include regional comparisons of key findings across the continent. From preliminary searches conducted, we anticipate the inclusion of a minimum of 10 eligible studies. Guidance on the conduct of narrative synthesis methodology in systematic reviews (by the ESRC Methods Programme) infers that seven studies are sufficient to acquire relatively rich data on factors influencing adolescent substance use.

Where applicable, a meta-analysis of quantitative studies will be conducted. This element is dependent on the type of resultant included studies. If within our final included studies, we have a subgroup of studies that are sufficiently homogeneous in terms of subjects involved and outcomes to provide a meaningful summary, we will conduct a meta-analysis [26]. Pooled effect sizes (e.g. proportions, odds ratios) of identified factors associated with substance use with 95% confidence intervals from included quantitative studies will be calculated using a random-effects model, based on expected heterogeneity of the included populations. Heterogeneity between the studies will be assessed using the *I*^2^ statistic, which describes the percentage of variation across studies that is due to heterogeneity rather than chance. *I*^2^ value greater than 50% will be considered as indicative of substantial heterogeneity. Summaries will be reported using effect sizes converted to Fisher’s *Z* transformed correlation coefficients and standardised mean differences.

For qualitative studies, a meta-synthesis will be conducted to re-interpret the meaning across the included qualitative articles and broaden the understanding around determinants and risk factors of adolescent substance use in Africa. This will involve aggregation or synthesis of findings to generate a set of statements that represent a category, through assembling the findings rated according to their quality, and categorizing these findings on the basis of similarity in meaning. These categories will then be subjected to a meta-synthesis in order to produce a single comprehensive set of synthesised findings that can be used as a basis for evidence-based practice. Where textual pooling is not possible, the findings will be presented in a narrative form.

## Discussion

This manuscript details a systematic review protocol to fill a gap in the literature around key determinants that lead to adolescent substance use in Africa. To our knowledge, this is the first systematic review on this topic in this context, and this evidence will be used to inform the development of effective prevention and treatment strategies, enhancing the achievement of SDGs 3.5 and 3.4 in the region of Africa [12, 23]. An accurate understanding of the determinants of substance use among adolescents is essential for the prevention and treatment of substance use and harmful use of alcohol, as enshrined in the Sustainable Development Goals (SDG3.5). The recent systematic review of substance use among adolescents in sub-Saharan Africa highlighted a prevalence of 41.6% [12]. Our study will build on this review with a focus on determinants and risk factors in Africa [9]. This will help to build on the knowledge of why existing prevention and treatment programmes for adolescents in the area of substance use work or not [5, 20]. This review will also help provide evidence to inform the development of interventions in regions where treatment and prevention programmes are lacking or limited [27].

### Limitations

There are some limitations we anticipate whilst performing our planned review. Potential issues at study and review levels include finding diversity in substance use types, study designs and outcomes, including country and/or region-specific trends among included articles [12]. Such heterogeneity in data makes it challenging for reviewers to accurately synthesis information and make comparisons. Although we plan to search for information across several databases and grey literature sources, missing some studies is inevitable. This may be a particular issue in the context of Africa where it is particularly challenging to conduct and publish research because of issues with access and collection of data, diversity of the region and a general lack of research infrastructure [28].

If there are any amendments made to this protocol when conducting the review, this will be outlined in PROSPERO and reported in the final outcome manuscript.

The results from this systematic review will be disseminated through conference presentations and publication in a peer-reviewed journal. We will also share review findings to non-academic audiences, particularly youth forums through collaborative work with key stakeholders like the National Youth Council of Malawi.

Evidence generated by this proposed systematic review can increase knowledge on why adolescents in Africa are driven to (harmfully) use substances [20]. This knowledge can inform further research looking to develop substance use prevention and treatment programmes that are cost-effective and geographically and culturally relevant [5, 20, 27]. Developing preventive interventions or models of care that are also cost-effective can significantly reduce burden on an overstretched health system particularly in resource-poor settings. On a wider community level, this research can be used to launch a platform for raising awareness of the multifaceted negative impact of substance misuse on the individual and society through community engagement and multi-disciplinary working, maximising grassroots dissemination and knowledge. This can consequently empower the youth to better manage their health and wellbeing independently.

## Supplementary Information

**Additional file 1.** PRISMA-P checklist.

**Additional file 2.** PubMed search strategy.

## Data Availability

Not applicable

## Abbreviations

DALYs: Disability-adjusted life years
SDG: Sustainable Development Goal
WHO: World Health Organization
AIM: African Index Medicus
CASP: Critical Appraisal Skills Programme
