# Supplementary material for: Determinants of adolescent substance use in Africa: a systematic review and meta-analysis protocol
Source: Syst Rev. 2021 Apr 27;10:125. doi: 10.1186/s13643-021-01680-y (PMC8080366; doi:10.1186/s13643-021-01680-y)
Supplement: Supplementary file 2 — Additional file 2. PubMed search strategy. [file 13643_2021_1680_MOESM2_ESM.docx]

| **PUBMED search strategy** |
| --- |
| ((adolescen*) OR teen* OR "young people" OR youth NOT (adults))  AND  ("substance use” OR “substance abuse” OR “substance misuse" OR “drug consumption” OR drug* OR alcohol* OR tobacco OR smok* OR marijuana OR cannab* OR heroin OR khat OR hookah OR shisha OR inhalant)  AND  (Africa* OR “sub-Saharan Africa”)  AND  (determinant OR factor* OR reason) |
